# 3-[1-(4-Isobutyl­phen­yl)eth­yl]-1*H*-1,2,4-triazole-5(4*H*)-thione

**DOI:** 10.1107/S1600536809006394

**Published:** 2009-02-28

**Authors:** Hoong-Kun Fun, Reza Kia, Robinson Jebas Samuel, K. V Sujith, B. Kalluraya

**Affiliations:** a X-ray Crystallography Unit, School of Physics, Universiti Sains Malaysia, 11800 USM, Penang, Malaysia; b Department of Studies in Chemistry, Mangalore University, Mangalagangotri, Mangalore 574 199, India

## Abstract

In the title compound, C_14_H_19_N_3_S, the dihedral angle between the mean planes of the five- and six-membered rings is 74.69 (4)°. Pairs of inter­molecular N—H⋯S inter­actions link neighbouring mol­ecules into dimers with *R*_2_^2^(8) ring motifs. These dimers are then linked together by the same type of inter­actions into an infinite one-dimensional chain along the *b* axis.

## Related literature

For hydrogen-bond motifs, see: Bernstein *et al.* (1995 ▶). For the biomedical applications compounds containing 1,2,4-triazole rings, see, for example: Shujuan *et al.* (2004 ▶); Clemons *et al.* (2004 ▶); Johnston *et al.* (2002 ▶); Wei *et al.* (2007 ▶). For the stability of the temperature controller used in the data collection, see: Cosier & Glazer (1986 ▶).
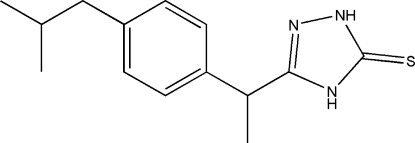


## Experimental

### 

#### Crystal data

C_14_H_19_N_3_S
*M* *_r_* = 261.38
Monoclinic, 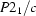

*a* = 12.0905 (2) Å
*b* = 8.4408 (1) Å
*c* = 14.3189 (2) Å
β = 98.365 (1)°
*V* = 1445.75 (4) Å^3^
*Z* = 4
Mo *K*α radiation
μ = 0.21 mm^−1^
*T* = 100 K
0.51 × 0.36 × 0.20 mm

#### Data collection

Bruker SMART APEXII CCD area-detector diffractometer
Absorption correction: multi-scan (**SADABS**; Bruker, 2005 ▶) *T*_min_ = 0.900, *T*_max_ = 0.959
27376 measured reflections
6797 independent reflections
5688 reflections with *I* > 2σ(*I*)
*R*_int_ = 0.027

#### Refinement

*R*[*F* ^2^ > 2σ(*F* ^2^)] = 0.038
*wR*(*F* ^2^) = 0.108
*S* = 1.03
6797 reflections
174 parameters
H atoms treated by a mixture of independent and constrained refinement
Δρ_max_ = 0.68 e Å^−3^
Δρ_min_ = −0.29 e Å^−3^

### 

Data collection: *APEX2* (Bruker, 2005 ▶); cell refinement: *SAINT* (Bruker, 2005 ▶); data reduction: *SAINT*; program(s) used to solve structure: *SHELXTL* (Sheldrick, 2008 ▶); program(s) used to refine structure: *SHELXTL*; molecular graphics: *SHELXTL*; software used to prepare material for publication: *SHELXTL* and *PLATON* (Spek, 2009 ▶).

## Supplementary Material

Crystal structure: contains datablocks global, I. DOI: 10.1107/S1600536809006394/cs2108sup1.cif

Structure factors: contains datablocks I. DOI: 10.1107/S1600536809006394/cs2108Isup2.hkl

Additional supplementary materials:  crystallographic information; 3D view; checkCIF report

## Figures and Tables

**Table 1:** Hydrogen-bond geometry (Å, °)

*D*—H⋯*A*	*D*—H	H⋯*A*	*D*⋯*A*	*D*—H⋯*A*
N2—H1*N*2⋯S1^i^	0.843 (14)	2.476 (14)	3.3150 (7)	173.9 (12)
N1—H1*N*1⋯S1^ii^	0.856 (15)	2.400 (15)	3.2549 (6)	176.4 (13)

Symmetry codes: (i) 
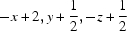
; (ii) 
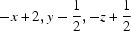
.

**Fig. 1.**
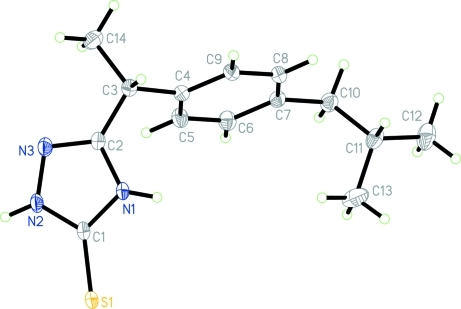
The molecular structure of (I) with atom labels and 50% probability ellipsoids for non-H atoms.

**Fig. 2.**
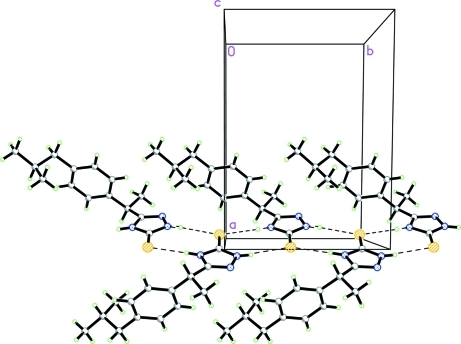
A crystal packing detail of (I), viewed down the (001) direction, showing 1-D infinite chains along (0 1 0) direction connected through intermolecular N—H···S contacts with R22(8) motifs, indicated in dashed lines.

## supplementary crystallographic information

### Comment

The 1,2,4-triazole nucleus has been incorporated into a wide variety of therapeutically interesting compounds. Several compounds containing 1,2,4-triazole rings are well known as drugs. For example, fluconazole is used as an antimicrobial drug (Shujuan *et al.*, 2004), while vorozole, letrozole and anastrozole are non-steroidal drugs used for the threatment of cancer (Clemons *et al.*, 2004) and loreclezole is used as an anticonvulsant (Johnston *et al.*, 2002). Similarly substituted derivatives of triazole possess comprehensive bioactivities such as antimicrobial, anti-inflammatory, analgesic, antihypertensive, anticonvulsant and antiviral activities (Wei *et al.*, 2007). Due to the progress that occurs in dealing with the chemistry of 1,2,4-triazoles as well as their biological activity, we synthesized and reported the crystal structure of the title compound.

The title compound, Fig. 1, comprises a single molcule in the asymmetric unit. The dihedral angle between the mean planes of the five- and six-membered rings is 74.69 (4)°. Pairs of intermolecular N—H···S interactions link neighbouring molecules into dimers with *R_2_^2^(8)* ring motifs (Bernstein *et al.*, 1995). These dimers are linked together into an infinite 1-D chains along the *b* axis.

### Experimental

A mixture of 2-[2-(4-isobutylphenyl)propanoyl]hydrazinecarbothioamide (0.01 mole) and 10% KOH (10 ml) was refluxed for 3 h. After the mixture was cooled to room temperature, it was then neutralized by the gradual addition of glacial acetic acid. The solid product obtained was collected by filtration, washed with ethanol and dried. It was then recrystallized using ethanol. Crystals suitable for *X*-ray analysis were obtained from ethanol solution by slow evaporation (Yield 71%; m.p. 473–474 K).

### Refinement

Hydrogen atoms bound to N1 and N2 were located from the difference Fourier map and refined freely; see Table. 1. The rest of the H atoms were positioned geometrically and refined with a riding model approximation with C—H = 0.95–1.00 Å and *U*_iso_(H) = 1.2 & 1.5 *U*_eq_(C). A rotating group model were used for the methyl groups. The highest peak (0.71 e. Å^-3^) is located 0.71 Å from C4 and the deepest hole (-0.29 e. Å^-3^) is located 1.07 Å from N2.

### Crystal data

**Table:**

C_14_H_19_N_3_S	*F*(000) = 560
*M**_r_* = 261.38	*D*_x_ = 1.201 Mg m^−^^3^
Monoclinic, *P*2_1_/*c*	Mo *K*α radiation, λ = 0.71073 Å
Hall symbol: -P 2ybc	Cell parameters from 9932 reflections
*a* = 12.0905 (2) Å	θ = 2.8–38.1°
*b* = 8.4408 (1) Å	µ = 0.21 mm^−^^1^
*c* = 14.3189 (2) Å	*T* = 100 K
β = 98.365 (1)°	Block, colourless
*V* = 1445.75 (4) Å^3^	0.51 × 0.36 × 0.20 mm
*Z* = 4	

### Data collection

**Table:**

Bruker SMART APEXII CCD area-detector diffractometer	6797 independent reflections
Radiation source: fine-focus sealed tube	5688 reflections with *I* > 2σ(*I*)
graphite	*R*_int_ = 0.027
φ and ω scans	θ_max_ = 36.0°, θ_min_ = 2.8°
Absorption correction: multi-scan (*SADABS*; Bruker, 2005)	*h* = −19→19
*T*_min_ = 0.900, *T*_max_ = 0.959	*k* = −13→13
27376 measured reflections	*l* = −23→23

### Refinement

**Table:**

Refinement on *F*^2^	Primary atom site location: structure-invariant direct methods
Least-squares matrix: full	Secondary atom site location: difference Fourier map
*R*[*F*^2^ > 2σ(*F*^2^)] = 0.038	Hydrogen site location: inferred from neighbouring sites
*wR*(*F*^2^) = 0.108	H atoms treated by a mixture of independent and constrained refinement
*S* = 1.03	*w* = 1/[σ^2^(*F*_o_^2^) + (0.059*P*)^2^ + 0.2413*P*] where *P* = (*F*_o_^2^ + 2*F*_c_^2^)/3
6797 reflections	(Δ/σ)_max_ = 0.001
174 parameters	Δρ_max_ = 0.68 e Å^−^^3^
0 restraints	Δρ_min_ = −0.29 e Å^−^^3^

### Special details

**Table:**

Experimental. The crystal was placed in the cold stream of an Oxford Cyrosystems Cobra open-flow nitrogen cryostat (Cosier & Glazer, 1986) operating at 100.0 (1) K.

### Fractional atomic coordinates and isotropic or equivalent isotropic displacement parameters (Å^2^)

**Table:**

	*x*	*y*	*z*	*U*_iso_*/*U*_eq_	
S1	1.028285 (17)	0.45561 (2)	0.304961 (13)	0.02021 (6)	
N1	0.93584 (6)	0.32637 (7)	0.13580 (4)	0.01752 (11)	
N2	0.93093 (6)	0.57817 (8)	0.13587 (5)	0.01827 (11)	
N3	0.88205 (6)	0.53370 (7)	0.04670 (5)	0.01893 (12)	
C1	0.96400 (6)	0.45407 (8)	0.19170 (5)	0.01660 (12)	
C2	0.88639 (6)	0.37897 (8)	0.04886 (5)	0.01656 (12)	
C3	0.84388 (6)	0.26915 (9)	−0.03029 (5)	0.01834 (13)	
H3A	0.9101	0.2190	−0.0529	0.022*	
C4	0.77413 (6)	0.13752 (9)	0.00436 (5)	0.01678 (12)	
C5	0.69207 (7)	0.17148 (9)	0.06075 (5)	0.02042 (13)	
H5A	0.6818	0.2775	0.0800	0.025*	
C6	0.62523 (7)	0.05153 (9)	0.08897 (6)	0.02088 (14)	
H6A	0.5701	0.0769	0.1276	0.025*	
C7	0.63774 (6)	−0.10552 (9)	0.06156 (5)	0.01780 (12)	
C8	0.72006 (7)	−0.13874 (9)	0.00540 (5)	0.01903 (13)	
H8A	0.7302	−0.2447	−0.0141	0.023*	
C9	0.78770 (7)	−0.01891 (9)	−0.02255 (5)	0.01853 (13)	
H9A	0.8436	−0.0444	−0.0603	0.022*	
C10	0.56594 (7)	−0.23645 (10)	0.09233 (6)	0.02152 (14)	
H10A	0.5276	−0.2910	0.0355	0.026*	
H10B	0.5077	−0.1887	0.1255	0.026*	
C11	0.63088 (7)	−0.35955 (10)	0.15737 (6)	0.02271 (14)	
H11A	0.6886	−0.4080	0.1228	0.027*	
C12	0.55200 (9)	−0.49136 (12)	0.18006 (8)	0.0324 (2)	
H12A	0.5165	−0.5405	0.1212	0.049*	
H12B	0.4944	−0.4465	0.2138	0.049*	
H12C	0.5946	−0.5715	0.2197	0.049*	
C13	0.69077 (9)	−0.28392 (15)	0.24717 (6)	0.0358 (2)	
H13A	0.7396	−0.1988	0.2307	0.054*	
H13B	0.7358	−0.3641	0.2848	0.054*	
H13C	0.6355	−0.2401	0.2838	0.054*	
C14	0.77858 (8)	0.35945 (11)	−0.11351 (6)	0.02686 (16)	
H14A	0.8260	0.4429	−0.1341	0.040*	
H14B	0.7118	0.4072	−0.0938	0.040*	
H14C	0.7562	0.2860	−0.1658	0.040*	
H1N2	0.9359 (11)	0.6745 (17)	0.1514 (9)	0.032 (3)*	
H1N1	0.9456 (11)	0.2302 (18)	0.1541 (10)	0.037 (4)*	

### Atomic displacement parameters (Å^2^)

**Table:**

	*U*^11^	*U*^22^	*U*^33^	*U*^12^	*U*^13^	*U*^23^
S1	0.02875 (10)	0.00993 (8)	0.02066 (9)	−0.00165 (6)	−0.00069 (7)	0.00020 (5)
N1	0.0229 (3)	0.0097 (2)	0.0194 (2)	0.0000 (2)	0.0011 (2)	0.00087 (18)
N2	0.0215 (3)	0.0097 (2)	0.0230 (3)	0.0005 (2)	0.0011 (2)	0.00087 (19)
N3	0.0215 (3)	0.0128 (2)	0.0220 (3)	0.0011 (2)	0.0017 (2)	0.0013 (2)
C1	0.0185 (3)	0.0102 (3)	0.0212 (3)	−0.0005 (2)	0.0032 (2)	0.0005 (2)
C2	0.0177 (3)	0.0129 (3)	0.0192 (3)	0.0001 (2)	0.0031 (2)	0.0015 (2)
C3	0.0216 (3)	0.0159 (3)	0.0177 (3)	−0.0010 (2)	0.0034 (2)	0.0005 (2)
C4	0.0192 (3)	0.0152 (3)	0.0156 (3)	−0.0007 (2)	0.0014 (2)	−0.0001 (2)
C5	0.0224 (3)	0.0163 (3)	0.0232 (3)	0.0012 (3)	0.0054 (2)	−0.0010 (2)
C6	0.0207 (3)	0.0191 (3)	0.0235 (3)	0.0010 (3)	0.0053 (3)	0.0000 (2)
C7	0.0183 (3)	0.0168 (3)	0.0174 (3)	−0.0009 (2)	−0.0006 (2)	0.0018 (2)
C8	0.0240 (3)	0.0149 (3)	0.0179 (3)	−0.0009 (3)	0.0020 (2)	−0.0008 (2)
C9	0.0230 (3)	0.0161 (3)	0.0168 (3)	−0.0004 (3)	0.0038 (2)	−0.0016 (2)
C10	0.0206 (3)	0.0206 (3)	0.0228 (3)	−0.0024 (3)	0.0012 (2)	0.0031 (3)
C11	0.0250 (3)	0.0214 (3)	0.0232 (3)	0.0027 (3)	0.0083 (3)	0.0058 (3)
C12	0.0369 (5)	0.0247 (4)	0.0393 (5)	0.0001 (4)	0.0183 (4)	0.0085 (4)
C13	0.0374 (5)	0.0468 (6)	0.0216 (4)	−0.0011 (4)	−0.0006 (3)	0.0079 (4)
C14	0.0317 (4)	0.0269 (4)	0.0208 (3)	−0.0036 (3)	−0.0005 (3)	0.0062 (3)

### Geometric parameters (Å, °)

**Table:**

S1—C1	1.6934 (8)	C7—C10	1.5101 (11)
N1—C1	1.3561 (9)	C8—C9	1.3954 (11)
N1—C2	1.3741 (9)	C8—H8A	0.9500
N1—H1N1	0.856 (15)	C9—H9A	0.9500
N2—C1	1.3429 (10)	C10—C11	1.5332 (11)
N2—N3	1.3786 (9)	C10—H10A	0.9900
N2—H1N2	0.843 (15)	C10—H10B	0.9900
N3—C2	1.3073 (10)	C11—C13	1.5212 (14)
C2—C3	1.4961 (10)	C11—C12	1.5307 (13)
C3—C4	1.5210 (10)	C11—H11A	1.0000
C3—C14	1.5332 (11)	C12—H12A	0.9800
C3—H3A	1.0000	C12—H12B	0.9800
C4—C9	1.3918 (10)	C12—H12C	0.9800
C4—C5	1.3970 (10)	C13—H13A	0.9800
C5—C6	1.3915 (11)	C13—H13B	0.9800
C5—H5A	0.9500	C13—H13C	0.9800
C6—C7	1.3969 (11)	C14—H14A	0.9800
C6—H6A	0.9500	C14—H14B	0.9800
C7—C8	1.3960 (10)	C14—H14C	0.9800
			
C1—N1—C2	108.46 (6)	C4—C9—C8	120.71 (7)
C1—N1—H1N1	124.2 (10)	C4—C9—H9A	119.6
C2—N1—H1N1	127.3 (10)	C8—C9—H9A	119.6
C1—N2—N3	112.91 (6)	C7—C10—C11	114.07 (6)
C1—N2—H1N2	126.2 (9)	C7—C10—H10A	108.7
N3—N2—H1N2	120.8 (9)	C11—C10—H10A	108.7
C2—N3—N2	103.88 (6)	C7—C10—H10B	108.7
N2—C1—N1	103.96 (6)	C11—C10—H10B	108.7
N2—C1—S1	128.29 (6)	H10A—C10—H10B	107.6
N1—C1—S1	127.75 (5)	C13—C11—C12	111.11 (7)
N3—C2—N1	110.79 (6)	C13—C11—C10	111.58 (8)
N3—C2—C3	126.37 (7)	C12—C11—C10	109.98 (7)
N1—C2—C3	122.85 (6)	C13—C11—H11A	108.0
C2—C3—C4	110.56 (6)	C12—C11—H11A	108.0
C2—C3—C14	111.23 (6)	C10—C11—H11A	108.0
C4—C3—C14	111.68 (6)	C11—C12—H12A	109.5
C2—C3—H3A	107.7	C11—C12—H12B	109.5
C4—C3—H3A	107.7	H12A—C12—H12B	109.5
C14—C3—H3A	107.7	C11—C12—H12C	109.5
C9—C4—C5	118.48 (7)	H12A—C12—H12C	109.5
C9—C4—C3	120.63 (6)	H12B—C12—H12C	109.5
C5—C4—C3	120.84 (7)	C11—C13—H13A	109.5
C6—C5—C4	120.65 (7)	C11—C13—H13B	109.5
C6—C5—H5A	119.7	H13A—C13—H13B	109.5
C4—C5—H5A	119.7	C11—C13—H13C	109.5
C5—C6—C7	121.18 (7)	H13A—C13—H13C	109.5
C5—C6—H6A	119.4	H13B—C13—H13C	109.5
C7—C6—H6A	119.4	C3—C14—H14A	109.5
C8—C7—C6	117.90 (7)	C3—C14—H14B	109.5
C8—C7—C10	120.62 (7)	H14A—C14—H14B	109.5
C6—C7—C10	121.48 (7)	C3—C14—H14C	109.5
C9—C8—C7	121.08 (7)	H14A—C14—H14C	109.5
C9—C8—H8A	119.5	H14B—C14—H14C	109.5
C7—C8—H8A	119.5		
			
C1—N2—N3—C2	−0.33 (8)	C14—C3—C4—C5	−77.63 (9)
N3—N2—C1—N1	0.44 (8)	C9—C4—C5—C6	−0.25 (11)
N3—N2—C1—S1	179.36 (5)	C3—C4—C5—C6	177.25 (7)
C2—N1—C1—N2	−0.36 (8)	C4—C5—C6—C7	−0.29 (12)
C2—N1—C1—S1	−179.29 (6)	C5—C6—C7—C8	0.43 (11)
N2—N3—C2—N1	0.08 (8)	C5—C6—C7—C10	179.67 (7)
N2—N3—C2—C3	179.70 (7)	C6—C7—C8—C9	−0.03 (11)
C1—N1—C2—N3	0.18 (9)	C10—C7—C8—C9	−179.28 (7)
C1—N1—C2—C3	−179.45 (6)	C5—C4—C9—C8	0.64 (11)
N3—C2—C3—C4	−132.25 (8)	C3—C4—C9—C8	−176.86 (7)
N1—C2—C3—C4	47.32 (9)	C7—C8—C9—C4	−0.51 (11)
N3—C2—C3—C14	−7.57 (10)	C8—C7—C10—C11	64.45 (9)
N1—C2—C3—C14	172.00 (7)	C6—C7—C10—C11	−114.77 (8)
C2—C3—C4—C9	−135.76 (7)	C7—C10—C11—C13	59.28 (9)
C14—C3—C4—C9	99.82 (8)	C7—C10—C11—C12	−176.94 (7)
C2—C3—C4—C5	46.79 (9)		

### Hydrogen-bond geometry (Å, °)

**Table:**

*D*—H···*A*	*D*—H	H···*A*	*D*···*A*	*D*—H···*A*
N2—H1N2···S1^i^	0.843 (14)	2.476 (14)	3.3150 (7)	173.9 (12)
N1—H1N1···S1^ii^	0.856 (15)	2.400 (15)	3.2549 (6)	176.4 (13)

Symmetry codes: (i) −*x*+2, *y*+1/2, −*z*+1/2; (ii) −*x*+2, *y*−1/2, −*z*+1/2.
